# Decrease in Healthcare Utilization and Costs for Opioid Users Following Residential Integrated Treatment for Co-Occurring Disorders

**DOI:** 10.3390/healthcare5030054

**Published:** 2017-09-07

**Authors:** Siobhan Morse, Brian E. Bride

**Affiliations:** 1 Foundations Recovery Network, Brentwood, TN 37027, USA; 2 School of Social Work, Georgia State University, Atlanta, GA 30302, USA; bbride@gsu.edu

**Keywords:** opioid use, co-occurring disorders, residential treatment, healthcare costs

## Abstract

Background: Opioid use results in higher healthcare utilization and costs, particularly among those with co-occurring mental health disorders. Presumably, effective treatment would result in a reduction in healthcare utilization and costs. To date, research has not examined this question. As such, the purpose of this study was to estimate and compare pre- and post-treatment healthcare utilization and costs for individuals receiving residential integrated treatment for co-occurring mental health and opioid use disorders. Methods: A single-group, repeated measures design was used to examine changes in pre- and post-treatment healthcare utilization and costs among a sample of individuals with co-occurring mental health and opioid use disorders who received residential, integrated treatment. Results: Significant reductions in emergency rooms visits, inpatient admissions, and resulting costs were observed in the six months following treatment. Conclusions: Residential, integrated treatment of co-occurring mental health and opioid use disorders can significantly decrease both utilization and cost of healthcare among opioid users with co-occurring mental health disorders.

## 1. Introduction

The marked increase in opioid consumption in the United States has been well chronicled. The US constitutes only 5% of the world population, yet Americans consume 80% of the world’s opioids and 99% of the worlds’ hydrocodone supply [1]. The Centers for Disease Control and Prevention (CDC) reports that four times as many prescription opioids were sold to pharmacies, hospitals, and doctors’ offices in the US in 2010 than in 1999 [2]. Between 1997 and 2007, average sales of prescription opioids in the US increased by more than 400% [1]. Further, the non-medical use of prescription opioid pain relievers has surpassed heroin as the most-commonly abused form of opioid and prescription pain relievers are the largest single category of illicit drug use other than marijuana [3].

The financial cost of opioid misuse, abuse, and dependence has been estimated upwards of $55 billion [4]. Chronic opioid use is linked to diminished quality of life and functionality and a greater risk of mortality than other illicit drugs [5,6,7]. The CDC reports that both consumption and overdose deaths have nearly quadrupled since 1999 [8]. The growing epidemic of opioid abuse in the United States not only dramatically affects patients’ lives, but it also impacts the use of healthcare services. Chronic drug users utilize 30% more emergency healthcare services than the general population [9]. Opioid users are at higher risk for hospitalization, making them a group of significant interest to healthcare providers and insurers. The rate of inpatient admissions that included a diagnosis of opioid overuse among adults aged 18 years and older increased more than 150% between 1993 and 2012 [10]. By 2012, the hospitalization rate for overuse of opioids, including dependence, abuse, poisoning and adverse effects alone had risen to 296 admissions per 100,000 people, up from 117 admissions per 100,000 in 1993 [11]. Opioid users often receive care in emergency rooms, one of the most expensive points of entry to the healthcare system [12]. Oxycodone-based medications were the pain relievers most commonly involved in emergency room visits (56.2 visits per 100,000) followed by hydrocodone, methadone and morphine followed (31.2, 24.3 and 12.3 visits per 100,000, respectively) [13]. Further, Wisniewski and colleagues (2008) found significant positive associations between prescribing opioids and emergency room visits [14].

Adults with mental health disorders are more likely to use opioids, have more severe health conditions, and thus have higher healthcare utilization rates than those without a mental health condition [15]. Because the evidence indicates that opioid use results in higher healthcare utilization, particularly among those with co-occurring mental health disorders, it would follow that successful treatment would result in a reduction in healthcare utilization and costs. However, we were surprisingly unable to locate any studies that documented such reductions. As such, the purpose of this exploratory study was to document and compare the utilization and costs of emergency room (ER) visits and hospital admissions to address medical, mental health, and substance use problems, respectively, in the six months preceding and subsequent to receiving residential integrated treatment for co-occurring substance use and mental disorders; and to estimate costs of ER visits and/or hospital admissions.

## 2. Materials and Methods

### 2.1. Data Collection

Data were collected in 2014 and 2015 from individuals who received treatment at four residential facilities operated by Foundations Recovery Network, the Addiction Services Division of Universal Health Services. These treatment facilities provide integrated substance abuse and mental health treatment services to patients drawn from across the United States and Canada. Admission criteria were based on medical necessity determined by insurance or other third-party payers and included substance use history and use patterns, psychiatric symptoms, previous treatment experience, and take into account medical conditions, and the social and legal consequences of the patient’s substance use. The first phase of residential treatment is assessment and stabilization. During this phase, patients receive an assessment by a multidisciplinary team, which provided the basis for an individual treatment plan to address substance use, psychiatric disorder, and medical and social service needs. A majority of opioid use disorder patients present with co-occurring disorders, with anxiety, depressive, and bipolar disorders being the most common. Stabilization for many patients who report opioid use in the 30 days prior to treatment includes detoxification with medications, including buprenorphine and other opioid detoxification medications and protocols. Co-occurring disorders were assessed and monitored over the course of treatment starting with initial screening, assessment, and psychiatric evaluation. Each participant was assigned to one of the program’s licensed clinicians who utilize the information gathered during screening and assessment to develop an initial treatment plan with the patient during the first week of treatment. Ongoing psychiatric and individual therapy sessions were provided in conjunction with weekly treatment team meetings to update each patient’s treatment plan. This process provided input from a multidisciplinary team of clinicians in order to thoroughly assess co-occurring disorders throughout treatment as symptoms may change or become clearer during the course of treatment. The typically expected length of stay was between 28 and 40 days, however, recommended treatment duration was individualized based on clinical assessment and medical necessity and averaged 31 days. Psychotropic medications may be prescribed, depending on disorder severity and treatment team recommendations. The use of buprenorphine-based medications is less common as a maintenance strategy in residential treatment, with most patients choosing alternative pathways such as abstinence and/or other types of medication assisted treatment.

Baseline data was obtained from face-to-face interviews at treatment entry. Telephone interviews at 30-days and 6-months post-discharge were used to collect follow-up data. A subset of items from the Treatment Services Review-Version 6 (TRS-6) [16] were utilized to measure healthcare utilization domains. Studies have demonstrated that the TSR-6 can obtain comparably accurate results when the reporting time frame is modified [17]. At intake, patients reported the number of emergency room visits in the previous six months for medical, mental health, and substance use problems, respectively. Patients also reported the number of nights they spent in the hospital for medical, mental health, and substance use problems, respectively. At the 30-day post-discharge interview, patients reported ER visits and hospital admissions since leaving residential treatment. At the six-month post-discharge interview, patients who did not participate in the 30-day follow up reported ER visits and hospital admissions since discharge. Patients who had completed the 30-day follow-up reported ER visits and hospital admissions since the last interview with us, and data from the 30-day and six-month interviews were summed within each category to obtain an estimate of ER visits and hospital admissions in the six months since discharge. Utilization costs for ER visits were calculated based upon an average cost of US$1579 per ER visit as estimated by the Agency for Healthcare Research and Quality [18]. Costs for hospital admissions were calculated based on an average cost of US$2212 per night as estimated by the Kaiser Family Foundation [19]. These cost estimates were used across all patients, although it should be noted that a small percentage (<8%) of patients treated were from Canada.

Because the purpose of this study was to examine healthcare utilization and costs among opioid users, we excluded those participants who indicated that they had not used heroin or prescription opioids, including methadone in a non-prescribed manner in the 30 days prior to treatment entry. All participants provided informed consent for inclusion prior to participating in the study. The study was conducted in accordance with the Declaration of Helsinki, and the protocol was approved by the Institutional Review Board of Foundations Recovery Network (FRN002013-01).

### 2.2. Planned Analyses

The first step in data analysis was to calculate descriptive statistics for the variables of interest, namely (1) the number of patients who reported ER visits and/or hospital admissions (2) the mean number of ER visits and/or hospital admissions, and (3) the estimated costs for ER visits and/or hospital admissions. For each of these variables statistics were calculated separately for the pre-treatment and post-treatment periods, and broken out for medical, mental health, and substance use problems, as well as for any of these reasons. The second step in data analysis was to calculate inferential statistics to determine if changes in ER and hospital utilization and cost rates differed between the pre-treatment and post-treatment periods. To test for differences between the pre-treatment and post-treatment periods in the proportion of patients who reported ER visits or hospital admissions, we utilized McNemar’s test, which is appropriate for use with paired nominal data. To test for differences in the number of ER visits and hospital admissions, we utilized paired samples t-tests. Lastly, to compare estimated costs of ER visits and hospital admissions, we utilized the paired samples sign test rather than the paired samples t-test due to concern the latter’s required assumption of normality was untenable due to highly skewed values in the sample.

## 3. Results

### 3.1. Sample Description

Of 2444 patients admitted between 1 January 2014 and 10 February 2015, 90% (*n* = 2200) consented to participate in the study and provided baseline data. Of those who completed data at intake, 66% (*n* = 1452) completed the one-month follow-up, and 64% (*n* = 1408) completed the six-month follow-up. For the purpose of these analyses, only patients (*n* = 967) who reported opioid use in the month prior to admission were included. Study participants were primarily White (93%, *n* = 899), non-Hispanic (94%, *n* = 909), and male (66%, *n* = 638), and ranged in age from 18 to 69, with a mean age of 29.3 (s.d. = 10.32). All participants reported opioid use prior to treatment entry with the following misuse patterns: prescription opioids only (42%, *n* = 410), heroin only (30%, *n* = 294), prescription opioids and heroin (19%, *n* = 187), prescription opioids, heroin and methadone (3%, *n* = 31), prescription opioids and methadone (3%, *n* = 29), heroin and methadone (9%), and methadone only (7%).

### 3.2. Healthcare Utilization

Columns 2 and 5 of Table 1 display the number of individuals who reported ER visits and/or inpatient admissions for medical, mental health, and substance use problems during the pre- and post-treatment periods. Columns 4 and 7 display the mean number of ER visits and inpatient nights among those who reported any utilization during the pre-and post-treatment periods.

In the six months prior to entering residential treatment for co-occurring substance use and mental health disorders, 28% of participants made at least one visit to the ER for medical problems, 12% for mental health problems, and 24% for substance use problems. In the six months following treatment, the proportion of patients visited an ER was 8% for medical problems, 3% for mental health problems, and 4% for substance use problems. These reductions were statistically significant at *p* < 0.001. The magnitude of the reductions were 61% for individuals who reported an ER visit for medical problems 69% for individuals who reported an ER visit for mental health problems, and 79% for individuals who reported an ER visit for substance use problems. Further, 64% (*n* = 616) of patients reported an ER visit for any reason in the pre-treatment period and 20% (*n* = 190) reported an ER visit for any reason in the post-treatment period (x^2^ = 386.04, *p* < 0.001).

In the six months prior to entering treatment, 13% of participants reported at least one inpatient day for medical problems, 9% for mental health problems, and 16% for substance use problems. In the six months following treatment, the proportion of patients who reported at least one inpatient day was 3% for medical problems, 2% for mental health problems, and 2% for substance use problems. These reductions were statistically significant at *p* < 0.001. The magnitude of the reductions were 62% for individuals who reported at least one inpatient day for medical problems 69% for individuals who reported at least one inpatient day for mental health problems, and 81% for individuals who reported at least one inpatient day for substance use problems. Further, 37% (*n* = 361) of patients reported an inpatient stay for any reason in the pre-treatment period and 11% (*n* = 102) reported an inpatient stay for any reason in the post-treatment period (x^2^ = 190.49, *p* < 0.001).

### 3.3. Healthcare Costs

Table 2 displays the estimated healthcare costs reported by participants for the six months prior to treatment and the six months following treatment, presented separately for medical, mental health, and substance use. Total pre-treatment ER costs were estimated at $1,650,055 while post-treatment ER costs were estimated to be $601,599; a 64% reduction. Comparison of total pre-treatment (mean = 1706, s.d. = 2974) and post-treatment (mean = 622, s.d. = 2608) ER costs was also significant (*t* = 9.337, *p* < 0.001). The average estimated cost per patient for ER visits ranged $360–$761 in the pre-treatment period and reduced to $185–$483 in the post-treatment period. A statistically significant reduction was demonstrated in each domain.

The average estimated cost per patient in the pre-treatment period for ER visits ranged from $299 for mental health reasons to $841 for medical reasons and reduced to $111 for mental health reasons and $364 for medical reasons in the post-treatment period. The average estimated cost of inpatient admissions in the pre-treatment period ranged from $830 for mental health reasons to $1091 for substance use reasons and reduced to $245 for mental health reasons and 464 for medical reasons in the post-treatment period. The estimated costs from ER visits and hospital admissions were significantly reduced (*p* < 0.05) in the six-month post-treatment period across all three domains. Total pre-treatment ER costs were estimated to be $1,650,055 while post-treatment inpatient costs were estimated at $601,599, a 64% reduction.

## 4. Discussion

As previously noted, that individuals with substance use and mental health disorders have higher rates of physical illness and not only utilize more health care than the general populations, but also utilize more costly levels of care such as emergency room visits at a higher rate. Utilizing the emergency room as entry level into care is not only more costly than a primary care visit, but also associated with delayed medical care which can further drive costs upward [20,21]. Although it is implicitly believed that successful treatment of co-occurring substance use and mental disorders results in decreased healthcare utilization and costs, the extant literature holds little empirical examination of this assumption. As such, this exploratory study was conducted to contribute to the nascent knowledge base in this area. Specifically, we sought to document and compare healthcare utilization and costs among individuals who received residential integrated treatment for co-occurring disorders. Results were consistent in demonstrating a reduction in utilization of ER visits and hospital admissions for medical, mental health, and substance use problems. In the six months prior to receiving treatment, patients reported a total of 2725 ER visits and/or hospital admissions. In the six months following admission, only 801 ER visits/hospital admissions were reported. Further, the estimated healthcare costs decreased from approximately $4.5 million in the six months prior to treatment to approximately $1.5 million in the six months following treatment. Thus in the aggregate, participants in this study experienced reduced healthcare utilization and costs after receiving treatment for co-occurring opioid use and mental health disorders. As one might anticipate, the greatest impact was in the use of the ER and hospital admissions for substance use problems. Patients who participated in integrated residential treatment for co-occurring substance use and mental health disorder were significantly less likely to seek substance use disorder care through the ER or hospital admission following treatment which resulted in a savings over pretreatment costs of $1,217,607 in the six months following treatment for these services alone. Significant savings were also noted for mental health disorder services of nearly $747,857 across the two levels of care. Medical savings were also significant at $956,556.

There are limitations to the conclusions that can be drawn from this study. First, the data are self-reported, rather than drawn from a comprehensive database of healthcare utilization and costs. Second, we focused on ER visits and hospital admissions, but excluded other healthcare services, such as primary care visits. Third, a comparison group was not utilized. Thus, we do not claim that residential integrated treatment results are better than other treatment approaches, such as outpatient treatment, treatment for substance use disorders only, or treatment for mental health disorders only. Fourth, our goal was to provide an overall snapshot of pre and post-treatment healthcare utilization and costs. Our results should not be construed to indicate that all individuals demonstrated improvement. Future research should be conducted to determine if there are differences in utilization based upon a variety of additional variables. Despite the limitations, these findings represent important new information regarding an issue lacking existing empirical data.

## Figures and Tables

**Table 1:** Number of participants with emergency room visits and inpatient admissions for physical, mental health, and substance use problems before and after treatment and mean number of ER visits/ and inpatient days (*n* = 967).

Problem Domain	Pre-Treatment			Post-Treatment		
	*n* (%)	Range	Mean (s.d.) *	*n* (%)	Range	Mean (s.d.) *
Emergency Room Visits						
Medical ^a,b^	272 (28%)	0–15	0.53 (1.26)	105 (8%)	0–30	0.23 (1.31)
Mental Health ^a,b^	115 (12%)	0–6	0.19 (0.64)	36 (3%)	0–15	0.07 (0.58)
Substance Use ^a,b^	229 (24%)	0–6	0.36 (0.80)	49 (4%)	0–7	0.09 (0.52)
TOTAL	457 (47%)	0–15	1.08 (1.88)	158 (16%)	0–30	0.39 (1.65)
Hospital Admissions						
Medical ^a,b^	124 (13%)	0–60	0.44 (2.64)	47 (3%)	0–32	0.21 (1.52)
Mental Health ^a,b^	87 (9%)	0–90	0.38 (3.36)	27 (2%)	0–14	0.11 (0.92)
Substance Use ^a,b^	150 (16%)	0–50	0.49 (2.57)	28 (2%)	0–15	0.11 (0.89)
TOTAL	256 (26%)	0–90	1.31 (5.53)	81 (8%)	0–32	0.43 (2.17)

Note: ^a^ McNemar’s test comparing pre-treatment and post-treatment utilization is significant at *p* < 0.05; ^b^ Paired samples *t*-test comparing pre-treatment and post treatment mean visits is significant at *p* < 0.05. * Mean ER visits/Inpatient admissions among patients are calculated based upon the total sample (*n* = 967).

**Table 2:** Estimated costs of ER visits and inpatient nights, pre-treatment and post-treatment (*n* = 967).

Problem Domain	Pre-Treatment			Post-Treatment		
	Visits/ Nights ^a^	Total Cost ($) ^b^	Mean (s.d.) Cost ($) ^c^	Visits/ Nights ^a^	Total Cost ($) ^b^	Mean (s.d.) Cost ($) ^c^
Emergency Room Visits						
Medical *	515	813,185	841 (1992)	223	352,117	364 (2072)
Mental Health *	318	288,957	299 (1014)	68	107,372	111 (922)
Substance Use *	570	547,913	567 (1257)	90	142,110	147 (815)
Total *	1458	1,650,055	1706 (2974)	381	601,599	622 (2608)
Hospital Admissions						
Medical *	427	944,524	977 (5842)	203	449,036	464 (3357)
Mental Health *	363	802,956	830 (7442)	107	236,684	245 (2029)
Substance Use *	477	1,055,124	1091 (5690)	110	243,320	252 (1969)
Total *	1267	2,802,604	2898 (12,223)	420	929,040	961 (4796)

Note: * *p* < 0.05. ^a^ Visits/Nights refers to the total number of ER visits or inpatient nights reported by participants. ^b^ Total cost refers to the total cost of ER visits and inpatient nights for each category. ^c^ Mean (s.d.) refers to the mean and standard deviation of costs per patient averaged across all patients.
